# Dissecting random and systematic differences between noisy composite data sets

**DOI:** 10.1107/S2059798317000699

**Published:** 2017-03-31

**Authors:** Kay Diederichs

**Affiliations:** a Department of Biology, University of Konstanz, Universitätsstrasse 19, 78457 Konstanz, Germany

**Keywords:** random and systematic error, correlation coefficient, eigenanalysis, sparse data, isomorphism, classification, dimensionality reduction

## Abstract

Composite data sets measured on different objects are usually affected by random errors, but may also be influenced by systematic (genuine) differences in the objects themselves, or the experimental conditions. If the individual measurements forming each data set are quantitative and approximately normally distributed, a correlation coefficient is often used to compare data sets. However, the relations between data sets are not obvious from the matrix of pairwise correlations since the numerical value of the correlation coefficient is lowered by both random and systematic differences between the data sets. This work presents a multidimensional scaling analysis of the pairwise correlation coefficients which places data sets into a unit sphere within low-dimensional space, at a position given by their CC* values [as defined by Karplus & Diederichs (2012 ▸), *Science*, **336**, 1030–1033] in the radial direction and by their systematic differences in one or more angular directions. This dimensionality reduction can not only be used for classification purposes, but also to derive data-set relations on a continuous scale. Projecting the arrangement of data sets onto the subspace spanned by systematic differences (the surface of a unit sphere) allows, irrespective of the random-error levels, the identification of clusters of closely related data sets. The method gains power with increasing numbers of data sets. It is illustrated with an example from low signal-to-noise ratio image processing, and an application in macromolecular crystallography is shown, but the approach is completely general and thus should be widely applicable.

## Abstract 2

A multidimensional scaling analysis of pairwise correlation coefficients is presented which positions data sets in a sphere with unit radius of an abstract, low-dimensional space at radii inversely proportional to their levels of random error and at spherical angles related to their mutual systematic differences. This reduction in dimensionality can not only be used for classification purposes, but also to derive data-set relations on a continuous scale.

## 1. Introduction   

Experimental data are a mixture of random and systematic components. Random components are generally referred to as ‘noise’. Systematic components are due to both genuine features of the systems or objects being studied, and to the specific way that the measurements are performed. Repeated measurements of data sets on the same objects may differ systematically if some experimental variables, such as the orientation or the composition of the objects, cannot be controlled. These systematic differences lead to systematic errors if they are not modelled. There is no known general procedure to distinguish between random and unknown (and therefore not modelled) systematic differences of data sets, which has given rise to a great number of specialized data-processing and classification procedures, each utilizing specific features of the system to analyze the data.

As an example, recent work in cryo-electron microscopy (cryo-EM) produces thousands of noisy images of molecular complexes that may be in slightly different conformations, or may have different compositions owing to the loss of subunits. Each image may be considered as a data set that can be compared with all others to establish its agreement and suitability for contributing to the molecular model of the complete complex. In favourable cases, one or a few reference data sets are available to decide how to classify each experimental data set. In the absence of reference data sets, a protocol may start from a small number of data sets to arrive at statistics and classifications describing all data sets. The danger associated with the extraction of such seed data sets is that they may lead to bias in the final results, as demonstrated by Shatsky *et al.* (2009 ▸) and Henderson (2013 ▸) for the case of low signal-to-noise cryo-EM images. More elaborate methods, which are well established in cryo-EM, use principal component analysis (PCA) followed by *k*-means clustering (Fu *et al.*, 2007 ▸) to extract common features, or a Bayesian maximum *a posteriori* (MAP) algorithm (Scheres, 2012 ▸). With good data, these methods perform adequately and have, for example, allowed the recent surge in structural results obtained by cryo-EM.

As another example, a complete crystallographic data set from a single large macromolecular crystal measured at a conventional X-ray source, a synchrotron beamline or a free-electron laser is usually comprised of thousands of unique intensity values from which the structure of the molecule can be derived. In the recently established field called ‘serial crystallography’, up to thousands of noisy partial data sets from tiny crystals may be measured and averaged to arrive at the final complete data used to calculate the macromolecular model. These data sets often do not only differ by random error, but also systematically, owing to, for example, differences in the unit-cell parameters, composition or conformation of the molecules that build the crystal lattice. Unfortunately, no current method is able to unambiguously classify these data sets according to their degree of relatedness, called ‘isomorphism’ in crystallography (and ‘homogeneity’ in many other fields). ‘Isomorphism’ in the strict sense of similarity of unit-cell parameters does not imply similarity of, for example, subunit composition and molecular conformation. A hierarchical clustering approach (Giordano *et al.*, 2012 ▸) based on pairwise correlation coefficients can be used, but correlation coefficients may be low owing to a high random error in the intensity values of one or both of the data sets being compared. This happens if the crystals are very small or the exposure is weak, and should not be taken as a sign of non-isomorphism. Current procedures are not satisfactory because they may miss systematic differences, and may thus lead to the inappropriate acceptance of data sets, or may discard valuable (but weak) data sets. By using a target function that measures the precision of the merged data, the latter problem may be avoided (Assmann *et al.*, 2016 ▸); however, the multitude of ways in which non-isomorphism between data sets may arise requires a multi-dimensional approach.

Here, an algorithm is described which separates the inter-data-set influences of random error from those arising from systematic differences, and reveals the relations between data sets represented as vectors in a low-dimensional space. It allows the identification of those data sets that differ only by random error, and could therefore, for example, be averaged to increase the signal-to-noise ratio. The averaged data set then corresponds to data from a single object, and reveals its properties more accurately than any single data set. On the other hand, groups of data sets that differ systematically, potentially corresponding to different specific combinations of object features, may be identified, clustered and analyzed.

## 2. Methods and theory   

Multidimensional scaling (MDS) is a family of methods that were developed more than 60 years ago (Torgerson, 1952 ▸). The purpose of MDS is to approximate a bivariate function *r* of *N* experimentally determined data sets **X** measured in a high-dimensional space with a bivariate function *l* of variables **x** in a low-dimensional space, with the intention of reducing the dimensionality of the experimental problem in order to help visualization, to allow clustering of the measurements and to perform further analyses. The basic MDS equation is
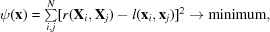
where *r* and *l* are bivariate functions of arguments defined in spaces of high (*M*) and low (*n*) dimensions, respectively (the terms used in the paper are summarized in Table 1 ▸). If *r* and *l* are metric data (*e.g.* distances), the method is called metric MDS; if *l* measures Euclidean distances, metric MDS is equivalent to a particular case of principal component analysis (PCA). Applications of metric MDS in the natural sciences are found, for example, in nuclear physics, cheminformatics and bioinformatics, such as in aspects of protein structure modelling (Chen, 2013 ▸) and detection of evolutionary relationships (Malaspinas *et al.*, 2014 ▸). Nonmetric MDS (typically with ordinal data) is mainly used in a social science context, such as in sociometry, market research, psychology, psychometrics and political science.

We recently described algorithms to resolve the twofold (or fourfold) indexing ambiguity occurring in serial crystallo­graphy, in certain space groups or for certain combinations of cell parameters (Brehm & Diederichs, 2014 ▸). These algorithms transform the (*N* ^2^ − *N*)/2 relations between *N* crystallo­graphic data sets, each given by the intensities of its unique reflections, into an *n* = 2 (or *n* = 4) dimensional space, thus allowing the visualization of inter-data-set relations.

We used the inter-data-set cross-correlation coefficients, calculated from intensities **X**_*i*_ of unique reflections (equivalent to greyscale values of pixels in images), as elements of the (real symmetric) matrix **r** = {*r*(**X**_*i*_, **X**_*j*_)} of dimensions *N* × *N*. The correlation coefficient was calculated only for those *i*, *j* pairs of data sets for which the number *M_ij_* of common unique reflections is high enough; we required at least five common reflections. The best algorithm of Brehm & Diederichs (2014 ▸) uses L-BFGS (Liu & Nocedal, 1989 ▸) to iteratively minimize, as a function of the vectors **x** *_i_* and **x** *_j_* representing the data sets in low-dimensional space (*n* = 2–4), the specific MDS equation
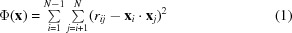
where the double summation extends over all pairs of data sets *i* and *j* with common reflections, *r*(**X**_*i*_, **X**_*j*_) = *r*_*ij*_ is the correlation coefficient between data sets *i* and *j*, and **x**_*i*_·**x**_*j*_ denotes the dot product of **x** *_i_* and **x** *_j_*. The properties of this algorithm are explained below and have, to my knowledge, not been described before; they were only understood after the publication of Brehm & Diederichs (2014 ▸).

For this algorithm’s choice of *r* and *l*, the least-squares solution may in principle be obtained algebraically by eigenanalysis of the matrix **r** = {*r*_*ij*_}. To this end, one writes **x** as a column vector composed of the *N* *n*-dimensional **x**_*i*_ and solves
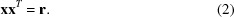



The *n* strongest eigenvalue/eigenvector pairs of **r** can be used for the construction of the *N* vectors **x**_*i*_ (Borg & Groenen, 2005 ▸). These lie in the unit sphere (sphere of radius one) within *n*-dimensional space. The *n*-dimensional solution thus exists; it is unique except for rotations of **x** around the origin and for changes of sign of one or more coordinate axes, since the dot product is invariant to both operations. However, when calculating the inter-data-set correlations, the *N* diagonal elements are not determined experimentally; therefore, instead of a direct algebraic solution of (2), an iterative least-squares solution of (1) may be obtained (Brehm & Diederichs, 2014 ▸). This procedure was found to be robust, for the specific problem that Brehm and Diederichs solved, even in the presence of a significant fraction of missing off-diagonal elements of **r** owing to low numbers *M_ij_* of unique reflections common to data sets *i* and *j*.

Common measurements (in crystallography, common unique reflections) can be thought of as establishing a direct connection by allowing the calculation of elements of **r** between data sets; data sets with no common measurements may still be connected indirectly through intervening data sets. A unique solution, consistent with the invariance properties of the dot product mentioned above, can be obtained from a sparse **r** matrix as long as each data set has *n* or more different direct or indirect connections to any other data set. This is because the least-squares solution **x** is robust with respect to the omission of specific *r_ij_* as long as the minimal connectivity of the data sets is maintained. As soon as the number of connections falls short of *n*, many additional solutions arise.

Generally, the value of *n* required to construct the **x**_*i*_ from eigenvalues/eigenvectors such that they approximate the *r_ij_* depends on the properties of the data sets. Since Pearson’s correlation coefficient can be written as a dot product (*i.e.* component-wise multiplication then summation) in *M_ij_*-dimensional space,
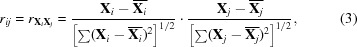
the dot product **x**_*i*_·**x**_*j*_ in *n*-dimensional space can be expected to approximate it adequately if *n* is high enough. This also applies to other types of correlation coefficients, such as Fisher’s (noncentred) product-moment correlation coefficient (Fisher, 1950 ▸) or Spearman’s rank correlation coefficient. With ideal, error-free data, the value of Φ is zero if the dot products **x**_*i*_·**x**_*j*_ exactly reproduce the correlation coefficients *r_ij_.* For a given case, the eigenvalues can be calculated after replacing (‘imputing’; Karhunen, 2011 ▸; Folch-Fortuny *et al.*, 2015 ▸) missing values in **r** = {*r_ij_*} by those computed, for example, from a least-squares solution. The minimum value of *n* can then be identified because it corresponds to the number of strong eigenvalues. This is still true if errors are present in the *r_ij_*; in this case, the *n* strongest eigenvalue/eigenvector pairs of **r** produce the least-squares solution of (2), and the remaining eigenvalues represent the noise.

Evidently, the *r_ij_* are not error-free as they are calculated from a finite-sized sample of noisy experimental data. This means that the properties of the solution of (1), as discussed below, are only approximately realised. However, the **x**_*i*_ vectors are better determined when the number *N* of experimental data sets increases, since the number (*N* ^2^ − *N*)/2 of matrix elements grows faster than that of the (*n* − 1)*N + 1 unknown **x**_*i*_ components. The accuracy of the components of **x**_*i*_ improves with the square root of *N*, in the same way as the average of *N* experimental measurements of a quantity approaches the population mean.

One property of the solution is particularly noteworthy: if several data sets **X**_*i*_ only differ in the amount of random error, then, provided that other data sets **Y** differ systematically and thus span the entire *n*-dimensional space, they are represented by vectors **x**_*i*_ in a one-dimensional subspace: a line through the origin. This is because the subset of equations involving the correlations of the **X**_*i*_ data sets can already be solved in *n* = 1 (see Appendix *A* [App appa]); this subset solution is only consistent with the properties of the dot product in *n* dimensions and the full system of simultaneous equations, which also includes the **Y** data sets, if the angles between the **x**_*i*_ vectors remain zero in *n*-dimensional space.

Vectors representing data sets consisting of random error have a length close to zero since these data sets yield a correlation close to zero with other data sets. On the other hand, the length of vectors cannot rise above one; vectors with a length of one represent the ‘proto’-types, *i.e.* the noise-free type of the data set; the length of a shorter vector with the same direction is given by its correlation with this prototypic data set. The cosines of angles between vectors **x**_*i*_ representing different prototypic data sets are given by the correlation coefficients between their data sets.

The lengths of vectors are thus inversely related to the amount of random error, and the angles between vectors represent genuine systematic differences.

An analytical relation for the signal-to-noise ratio of a given data set and the vector length follows from recent insight (Karplus & Diederichs, 2012 ▸) which defines a correlation with prototypic (‘true’) data as the quantity CC_true_, and finds that CC_true_ can be estimated by CC*, an analytical function of the intra-data-set correlation coefficient CC_1/2_, which in turn can be calculated from repeated measurements that are part of the same data set. This means that the value of CC* of a data set **X**_*i*_ is an estimate of CC_true_, the length of the vector **x**_*i*_ representing **X**_*i*_; its prototypic data set resides on the sphere, at the same spherical angles as those of **x** *_i_*.

As was also shown (Karplus & Diederichs, 2015 ▸), another relation exists between CC_1/2_ and the signal-to-noise ratio when the signal is normally distributed. The two equations linking CC_1/2_ to CC* and the signal-to-noise ratio 〈*I*〉/〈σ〉, respectively, can then be combined as
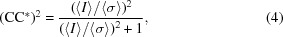
which shows that for very low 〈*I*〉/〈σ〉 CC* has a slightly lower numerical value than 〈*I*〉/〈σ〉, and approaches one if 〈*I*〉/〈σ〉 approaches infinity. Similar equations exist for the (crystallo­graphic) case of signal following an acentric or centric Wilson distribution.

The relevance of this connection is fourfold. Firstly, the lengths of the vectors obtained by solving (1) can be interpreted as CC* values that are analytically related to CC_1/2_, which measures the internal consistency of each data set. Secondly, a noise level can be assigned to each data set (through its vector length) if it is not already provided by the experimental procedure, and data sets in or close to a one-dimensional subspace (a direction in *n*-dimensional space) can properly be averaged, with weights according to their 〈*I*〉/〈σ〉 ratios. Thirdly, a CC* value can be assigned to a data set even if its internal consistency cannot be calculated owing to a lack of repeated measurements. Finally, and maybe most importantly, the correlation coefficient *r_ij_* between two data sets *i* and *j* can be expressed as 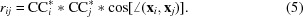



Thus, if 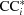 and 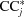 are available through calculation of their CC_1/2_ values, as is often the case for crystallographic data, the maximum possible correlation coefficient between the data sets is given by the product of their CC* values, and any reduction that the actual *r_ij_* displays must be owing to systematic error. The angle corresponding to the degree of their non-isomorphism may then be readily calculated from (5). This relation was unknown at the introduction of the CC* concept (Karplus & Diederichs, 2012 ▸), and highlights its utility.

## 3. Results and discussion   

**Figure 1:**
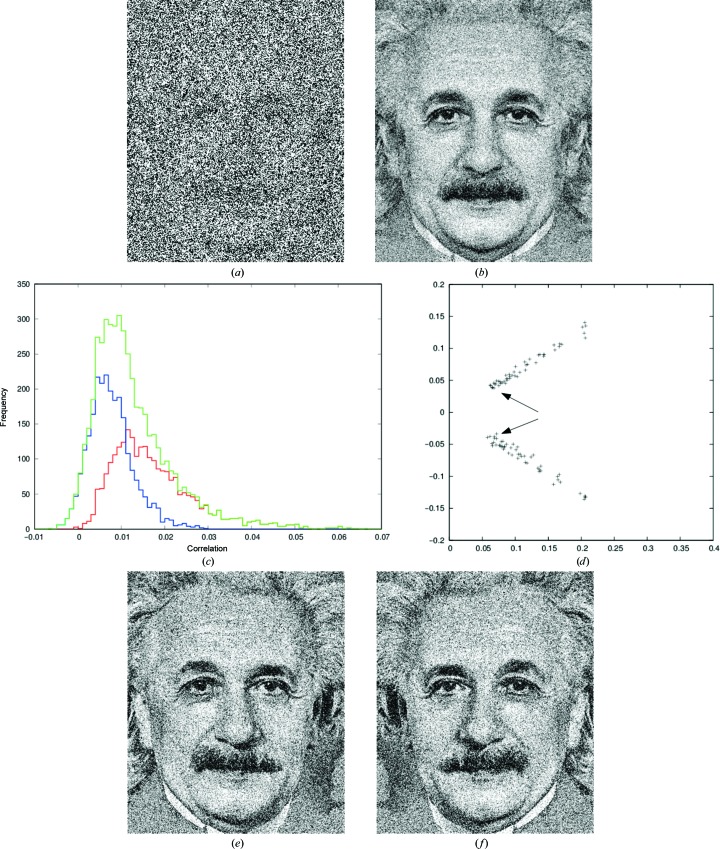
Portrait of A. Einstein (Wikimedia). (*a*) Example of portrait with added noise; the signal-to-noise ratio is 1:9. (*b*) Symmetric result of averaging of noisy images and mirror images. (*c*) Histograms of correlation coefficients (red, between images of the same type; blue, between images of different types; green, sum of both histograms). (*d*) Result of two-dimensional analysis: each cross represents one image. Arrows point to images with a 1:13 signal-to-noise ratio. The axes are unitless; only the relevant area of the possible range (a circle with radius 1) is shown. The angle between the two prototypic directions is 65°; its cosine agrees with the correlation of 0.43 between the image and its mirror. (*e*) The result of averaging the 50 noisy original images; the overall noise level is reduced by averaging. (*f*) as (*e*) but for the 50 noisy mirror images

These properties are first illustrated with a simple synthetic example which was generated for this work and is related to image analysis at weak signal-to-noise ratios. The face of Albert Einstein was extracted from a photograph taken by Sophie Delar in 1935, and 50 noisy synthetic images were computed by adding ten levels (resulting in signal-to-noise ratios of 1:4, 1:5, …, up to 1:13) of Gaussian noise with mean zero to the original pixel values. This procedure was repeated with the mirror image of Einstein; an example of an image with a signal-to-noise ratio of 1:9 is shown in Fig. 1 ▸(*a*).

The resulting collection of images mimics a situation, for example, in microscopy where two similar types of objects are imaged multiple times at such a low signal-to-noise ratio that the types of objects cannot be distinguished directly from the images, or where an object with an approximate twofold symmetry is imaged from its two different sides.

The problem in such experiments is to realise that different groups of objects exist and to correctly assign the noisy images such that they can be averaged within their respective group. If no assignment is possible and all images are averaged, the information about the difference between the objects is lost and a symmetric image results (Fig. 1 ▸ *b*). For the synthetic example data, the inter-group and intra-group correlation histograms (Fig. 1 ▸ *c*) were calculated and found to overlap. Thus, a separation of inter-group and intra-group correlation coefficients would not be possible because the images are too noisy to separate them into groups based on simple statistics (the two classes of images would however be separable with more elaborate classification methods).

The solution of (1) with the example data is shown in Fig. 1 ▸(*d*). The vectors representing the two groups of different images are well separated and easily distinguishable, as they are located in one-dimensional subspaces of the two-dimensional diagram, as expected from the properties of the method outlined above. The cosine of the angle between the two groups of vectors indeed agrees numerically with the correlation coefficient between the original image and its mirror image. Consistent with the theory outlined above and according to (4), Fig. 1 ▸(*d*) shows that the lengths of the vectors are related to their signal-to-noise ratios, with short vectors for the weakest signal-to-noise ratios and the longest vectors for the highest (but at 1:4 still weak) signal-to-noise ratios.

This example not only achieves classification of images, but also reveals their relations on a continuous scale: for systematically different images, the angle between the vectors representing them, and for images that differ only randomly, by the vector lengths.

It is noteworthy that the procedure achieves the clustering of similar images without requiring any initial or seed images, and could, for example, be used for the clustering of projections of three-dimensional objects rotated and translated in space. If several subunit compositions or conformational substates of the imaged object exist, the dimensionality, when calculating the solution of (1), should be adjusted according to the number of strong eigenvalue/eigenvector pairs of the **r** matrix.

The procedure is fast since only a small number of eigenvalues/eigenvectors are required, and the eigenanalysis does not depend on the number *M* of image pixels from which the correlation coefficients are calculated. It may provide unbiased seeds calculated from averages of images corresponding to the same orientation, the same composition and the same conformation for back-projection procedures that re-constitute the three-dimensional object.

The advantage of the method is that no prior information about the type of non-isomorphism (inhomogeneity) is required. A possible disadvantage is that a particular dimension of the solution is not directly interpretable as a particular object property, and data sets with strong components in that particular dimension first have to be averaged and analyzed to understand the ‘meaning’ of that dimension. The latter task is simple in the illustrative example just given, but may be nontrivial in other applications, such as in the next example.

In the following, the application of the method to experimental data obtained from an X-ray free-electron laser (XFEL) is shown. The femtosecond pulses produced by these devices are, despite their short duration, so intense that they allow only a single snapshot of the diffracted X-rays from a crystal to be measured before the crystal explodes. Each snapshot yields a partial data set, and many partial data sets have to be merged and averaged into a complete data set that can be analyzed to obtain the structure of the macromolecule. Owing to a lack of analysis methods that can cope with the high noise level of the individual partial data sets, it is currently unknown which range of macromolecular conformations the crystals sample, and whether structural insight about molecular conformations can be captured and extracted from these data. The usual current procedure is to merge and average all partial data sets and thus to arrive at the averaged structure, similar to what was seen in the previous example.

**Figure 2:**
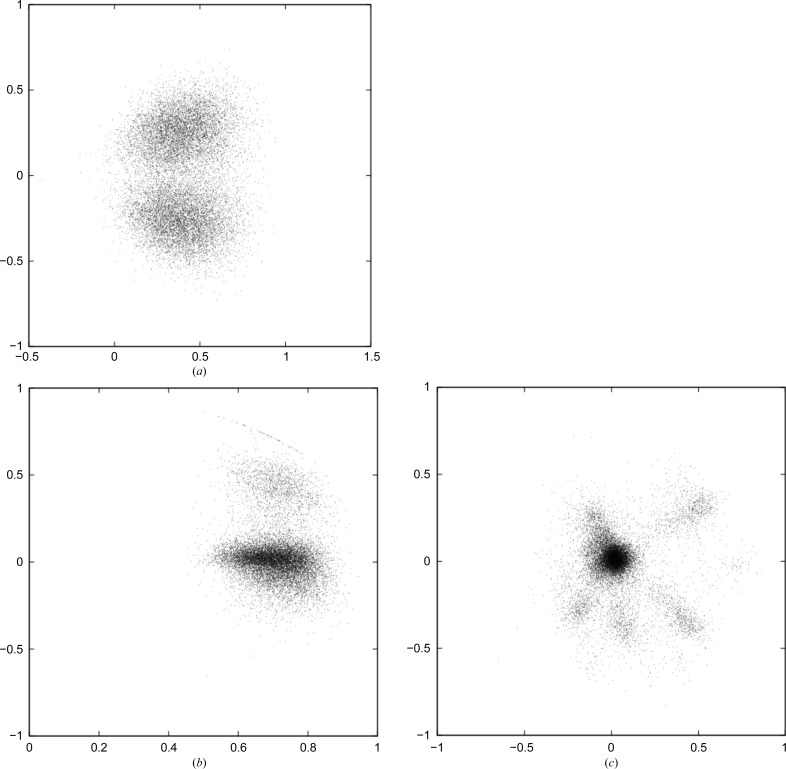
(*a*) Analysis of original photosystem I XFEL data shows two clusters corresponding to the two possible indexing modes. (*b*) Analysis of properly indexed photosystem I XFEL data; projection on the *xy* plane. (*c*) Analysis of properly indexed photosystem I XFEL data; projection on the *yz* plane.

The specific data used for analysis are from a large membrane-protein complex, photosystem I (36 proteins, 381 cofactors), and represent the first XFEL data obtained from a macromolecule (Chapman *et al.*, 2011 ▸). The indexing ambiguity present in the space group of these crystals was resolved (Brehm & Diederichs, 2014 ▸) by solving (1). In that work, the distribution of vectors in two-dimensional space (Fig. 2 ▸ *a*) was used for a binary decision for each of the 15 445 data sets (maximum resolution 8.7 Å) that implied either re-indexing or retaining the original reflection indices. Consistent with the theory outlined above, the angle between the centres of the clouds representing the two indexing choices is close to 90°; it is actually less because the overall falloff of intensities in the two indexing modes is the same, which results in a slightly positive correlation of their intensities even if the indexing mode differs.

For this work, these data were first re-indexed according to the results of Brehm & Diederichs (2014 ▸), as represented in Fig. 2 ▸(*a*), and subsequently subjected to the method described above. If the indexing mode were the only type of systematic difference between the data sets, the data sets would only differ in random error and would give a single strong eigenvalue in an *n* = 1 analysis. However, the calculation yielded eigenvalues beyond the first one that were more than ten times higher than the root-mean-square value of the remaining eigenvalues, and the calculation was therefore repeated with larger values of *n*. The results for *n* = 3 are shown as projections in the *xy* plane (Fig. 2 ▸ *b*) as well as in the *yz* plane (Fig. 2 ▸ *c*). Fig. 2 ▸(*b*) shows, as expected, the majority of data sets (∼10 000) in a single cluster elongated along its axial direction which nearly coincides with the *x* axis, but reveals a significant number of data sets that do not belong to it. Their locations in three-dimensional space are visible in the *yz* projection (Fig. 2 ▸ *c*). Here, projecting along the *x* axis on the subspace of systematic differences, the elongated main cluster appears at the strongly populated centre, and is surrounded by five smaller clusters of <1000 data sets each and one small cluster of a few hundred data sets. Each of these additional clusters represents a type of data set that differs systematically from the type represented by the main cluster.

It can be assumed that some of the systematically different types correspond to different contents of the crystals or conformations of its constituents; other types may result from peculiarities of the measurement or from artifacts of the software used to process the data. Analysis of the smaller clusters would give important insight about this experiment and its biological objects, but needs additional information that is not available for this experiment. The most straightforward use of the result consists of treating the data sets outside the main cloud as outliers, and thus merging and averaging only those data sets that have small systematic differences and differ mainly in their random error. This procedure is aided by the knowledge of the average noise levels of the data sets, given by the lengths of their vectors CC* and (4). However, such an analysis is beyond the scope of this work, which focuses on introducing the method and revealing its properties.

## 4. Summary   

The analysis demonstrated here has the novel and fundamental ability to explicitly separate random and systematic components of differences between data sets. Specifically, each vector representing a data set is placed within the *n*-dimensional unit sphere such that its length estimates CC_true_ (Karplus & Diederichs, 2012 ▸), the correlation to the ‘prototypic data set’ at the same spherical angles on the surface of the sphere. Furthermore, the *n* − 1 spherical angles describing the direction of the radius vector parameterize the *n* − 1 orthogonal ways in which the data sets differ systematically.

One advantage of the method is that the systematic ways in which the data sets differ do not have to be known beforehand; the number of dimensions required to describe the systematic differences is a result of the analysis. A potential disadvantage is that any particular dimension of the solution does not immediately reveal the object property that it parameterizes; this requires additional downstream analyses.

In structural biology, the procedure allows the solution of, for example, the classification problems associated with the averaging of data sets in the presence of inhomogeneity (‘non-isomorphism’ in crystallography), and links measures of the internal agreement of data sets (CC_1/2_) and a recently introduced correlation (CC*; Karplus & Diederichs, 2012 ▸) with a prototypic (usually not accessible ideal) data set to random and systematic differences of data sets as assessed by pairwise correlation coefficients. The method achieves dimensionality reduction and allows the prediction of, as in the case of XFEL data, the relations between data sets that have no common measurements and thus cannot be directly compared. It offers a way to extract structural information from noisy data sets, for example, in serial crystallography, and potentially in imaging techniques, that go beyond or at least complement the methods that are currently available. ‘No quantity has been more characteristic of biometrical work than the correlation coefficient, and no method has been applied to such various data as the method of correlation’ (Fisher, 1950 ▸). Since correlation coefficients are used ubiquitously in all sciences, many applications of the method are conceivable beyond structural biology. For example, correlations between physiological data of patients may be analyzed to identify prototypes of diseases, correlations of stock prices may yield improved portfolio choices, or communication events may be analyzed to reveal clusters to which the participants belong, and their relations. These and many more kinds of data can be analyzed, if correlations can be calculated, within the general framework outlined here, to classify and quantify systematic effects, and to separate them from random noise.

## Figures and Tables

**Table 1:** Terms used in this paper

Term	Meaning	Example(s)
*M*	Number of value pairs for correlation-coefficient calculation between data sets (*M_ij_* if specific for each *i*, *j* pair of data sets)	Number of unique reflections common to two data sets; number of image pixels within a mask
*N*	Number of experiments	Number of data sets; number of images
*n*	Dimension of reduced space	2
*r*	Bivariate scalar function of data sets *i* and *j*	Correlation coefficient between data sets with *M*_*ij*_ common unique reflections
*l*	Bivariate scalar function of the representation of data sets *i* and *j*	Scalar product in *n*-dimensional space
**X** *_i_*	Experimental data of data set *i*	Reflection intensities of data set *i*; pixel values of image *i*
**x** *_i_*	Representation of data set *i* in *n*-dimensional space (unit sphere)	Points in plane representing images (Fig. 1 ▸) or data sets (Fig. 2 ▸)
CC	Correlation coefficient	CC_1/2_, CC*
σ	Estimated standard deviation	Estimated error of intensity value
Systematic difference	Changes of experimental result owing to features of particular object; may be common to some data sets. Systematic differences lead to non-isomorphism/inhomogeneity.	Different conformation of molecule leading to different image or diffraction
Random error/difference	Unpredictable change of experimental result arising from effects that cannot be controlled by the experimenter and are unrelated to changes in other measurements of the same data set or in other data sets	Poisson statistics in photon-counting experiments; electronic noise in measurement apparatus; statistical variation within samples drawn from a homogeneous population

## Appendix A Correlation of data sets that differ only by random error

The definition of Pearson’s correlation coefficient is

where the summation index is left out. Suppose that **T** represents the noise-free data of a prototypical object. Further suppose that the experimental data sets **X**_*i*_ and **X** *_j_* differ from **T** by unrelated error terms **∊**_*i*_ and **∊**_*j*_ with zero mean, respectively. They may additionally differ by some scale factor, but since the correlation coefficient is invariant to scale factors, these scale factors can be taken to be 1. We may write *r_ij_* as

with **Y**_*i*_ = **X**_*i*_ − , **Y**_*j*_ = **X**_*j*_ − . Then, with **τ** = **T** − , we obtain **Y**_*i*_ = **τ** + **∊**_*i*_ and **Y**_*j*_ = **τ** + **∊**_*j*_. It follows that

since the sum over the terms **τ∊**_*i*_, **τ∊**_*j*_ and **∊**_*i*_ **∊**_*j*_ can be neglected. With the same argument, the last line can be identified as the product of Pearson’s correlation coefficients of **X**_*i*_ and **X**_*j*_, respectively, with **T**.

This means that the correlation coefficient of two multidimensional data vectors that differ only by unrelated random noise from a third data vector (**T**) can be represented as the product of the two correlation coefficients against the third data vector. This result, although easy to derive, appears to be difficult to find in the statistical literature; it was also used to derive equation (15) of Read & McCoy (2016 ▸).
